# P03-17 Evaluation of an asset-based, participatory physical activity promotion intervention in Dutch adolescents: a parallel group randomized trial

**DOI:** 10.1093/eurpub/ckac095.053

**Published:** 2022-08-29

**Authors:** Huib van de Kop, Anne den Uil, Joske Nauta, Huub Toussaint, Vincent Busch, Arnoud Verhoeff, Mirka Janssen

**Affiliations:** 1 Faculty of Sports and Nutrition, Amsterdam University of Applied Sciences, Amsterdam, The Netherlands; 2 Sarphati Amsterdam, Public Health Service (GGD), Amsterdam, Netherlands, Amsterdam, The Netherlands; 3 Faculty of Social and Behavioural Sciences, University of Amsterdam, Amsterdam, Netherlands, Amsterdam, The Netherlands

**Keywords:** Physical activity, physical fitness, adolescent, school-based intervention

## Abstract

**Background:**

Adolescents tend to be less physically active and thus become more vulnerable to health risks. Engaging adolescents becoming agents of their active lifestyle could potentially catalyze the impact of interventions. Therefore, the effects of an asset-based physical activity promotion intervention on the physical activity behavior and physical fitness of pre-vocational students was evaluated, taking into account the extent to which the students were involved in the design and implementation of the interventions.

**Methods:**

This randomized controlled trial included 2286 prevocational students divided between an intervention and control group. Innovative triple-i interactive research methods were used to develop and implement tailor-made interventions in co-creation with students and teachers. Self- reported physical activity behavior and direct assessments of physical fitness levels were determined on baseline and two annual follow-ups. Student involvement was determined by validated questionnaires. Generalized estimating equations were performed to determine intervention effects over time. Sensitivity analyses were conducted to evaluate the effects of the extent of student involvement.

**Results:**

No intervention effects were found for total physical activity level, screen time, active transport and sports activities (CI includes zero; p > 0.05). Positive effects of the intervention were found for the long jump (β = 3.44; CI = 1.29:5.58; p = 0.01), the handgrip strength (β = 0.55; CI = 0.02:1,08; p = 0.04), the shuttle run test (β = 0.28; CI = 0.02:0.54; p = 0.03) and the sum of skinfolds (β=-0.08; CI=-0.11:-0.06; p = 0.01). Subgroup analyses on the extent of student involvement showed mixed results. Students at intervention schools where students involvement was more successful showed no alterations in physical activity levels and higher scores on shuttle run scores (β = 0.58;CI=0.29:0.87; p = 0.00) compared to controls. Students at intervention schools that were less involved, showed higher scores on long jump (β = 7.77; CI = 4.78:10.76; p = 0.00), grip strength (β = 1.34; CI = 0.61:2.06; p = 0.00), and sum of skinfolds (β=-0.15; CI=-0.19:-0.10; p = 0.00).

**Conclusions:**

The participatory physical activity intervention in this study does not change physical activity level but does improve some elements of physical fitness. The research methods used in this study offers a protocol to align intervention plans to the students' assets by co-creation which is feasible to use in the school context.

